# BCI to Potentially Enhance Streaming Images to a VR Headset by Predicting Head Rotation

**DOI:** 10.3389/fnhum.2018.00420

**Published:** 2018-10-16

**Authors:** Anne-Marie Brouwer, Jasper van der Waa, Hans Stokking

**Affiliations:** ^1^Department of Perceptual and Cognitive Systems, Netherlands Organization for Applied Scientific Research (TNO), Soesterberg, Netherlands; ^2^Department of Media Networking, Netherlands Organization for Applied Scientific Research (TNO), Den Haag, Netherlands

**Keywords:** EEG, brain computer interface, neuroadaptive technology, virtual reality, head mounted display, head rotation, movement prediction, applied neuroscience

## Abstract

While numerous studies show that brain signals contain information about an individual’s current state that are potentially valuable for smoothing man–machine interfaces, this has not yet lead to the use of brain computer interfaces (BCI) in daily life. One of the main challenges is the common requirement of personal data that is correctly labeled concerning the state of interest in order to train a model, where this trained model is not guaranteed to generalize across time and context. Another challenge is the requirement to wear electrodes on the head. We here propose a BCI that can tackle these issues and may be a promising case for BCI research and application in everyday life. The BCI uses EEG signals to predict head rotation in order to improve images presented in a virtual reality (VR) headset. When presenting a 360° video to a headset, field-of-view approaches only stream the content that is in the current field of view and leave out the rest. When the user rotates the head, other content parts need to be made available soon enough to go unnoticed by the user, which is problematic given the available bandwidth. By predicting head rotation, the content parts adjacent to the currently viewed part could be retrieved in time for display when the rotation actually takes place. We here studied whether head rotations can be predicted on the basis of EEG sensor data and if so, whether application of such predictions could be applied to improve display of streaming images. Eleven participants generated left- and rightward head rotations while head movements were recorded using the headsets motion sensing system and EEG. We trained neural network models to distinguish EEG epochs preceding rightward, leftward, and no rotation. Applying these models to streaming EEG data that was withheld from the training showed that 400 ms before rotation onset, the probability “no rotation” started to decrease and the probabilities of an upcoming right- or leftward rotation started to diverge in the correct direction. In the proposed BCI scenario, users already wear a device on their head allowing for integrated EEG sensors. Moreover, it is possible to acquire accurately labeled training data on the fly, and continuously monitor and improve the model’s performance. The BCI can be harnessed if it will improve imagery and therewith enhance immersive experience.

## Introduction

Real time monitoring of cognitive and affective processes using brain signals could be potentially useful in a range of everyday applications such as real time adaptation of automated systems to fit the current state of a particular individual (Parasuraman and Rizzo, 2007). Such neuroergonomic applications are referred to as (passive) brain computer interfaces (BCI) (Zander et al., 2008) or neuroadaptive technology (Zander et al., 2016). While impressive progress has been made in the field, we think it is still difficult to pinpoint concrete applications where these estimates from brain signals could, in the near term, support the user enough to justify wearing electrodes. There are several reasons for this (Brouwer et al., 2015). One is that in many cases, other measures of cognitive and affective state that are available, or can be extracted, are more reliable and/or easier to interpret (such as user performance, behavioral measures, and explicit user input). While these measures may suffer from other specific disadvantages, neuroadaptive technology will only flourish in a scenario where even a limited reliability can be exploited such that the user benefits outweigh the costs. Perhaps even more important is the problem of acquiring data to train the BCI system. Such data for training should preferably be collected for the individual that is going to use the BCI and under the same (real life) conditions as where the BCI system is to be used, and updated regularly. Correct labels (i.e., the “true” cognitive or affective state that goes with a data interval) are, especially under real life conditions, often difficult to acquire. We here present a possible BCI application that can be envisioned to provide added value relatively soon, based on currently existing methods and equipment, since it is a system that automatically collects personal, correctly labeled data without user effort, it can validate itself on the fly and gradually improve the man–machine interaction even when the accuracy of its predictions is not perfect. Errors do not have dangerous consequences and users do not need to put extra equipment on the head. Also, it is likely that at least in some applications, brain signals are more informative than other possible sources of information. The proposed BCI application is the prediction of head movements in order to reduce delays in images presented in VR headsets. Especially when presenting streaming video data, choices in usage of bandwidth have to be such that image resolution is sacrificed to reducing delays in the viewed image when the head moves. This trade-off could be chosen more optimally if we would know whether it is likely the head is going to rotate, and if so, in which direction. These predictions may be made using EEG signals.

The main method for streaming video to VR headsets is to stream an entire 360° video to the receiver, possibly in 3D. Streaming high-quality (possibly 3D) panoramic views easily require tens or hundreds of Mbps, even with modern video encoding techniques (Schilt et al., 2016), posing a high-computational load and high-power consumption, which are disadvantageous for many devices. As VR rendering devices frequently stream the video stream via a bandwidth constrained network, for example, a digital subscriber line (DSL), wireless LAN (WLAN), or mobile connection (e.g., UMTS or LTE), the bandwidth requirements result in low-video quality or streaming is not even possible at all. To improve such streaming, a number of methods have been developed to use the bandwidth only (or mostly) for the part that is currently being looked at; only this part needs to be displayed and sent to the VR headset. These methods are called field-of-view (FoV) based streaming approaches (Brandenburg et al., 2017; Podborski et al., 2017).

**FIGURE 1:**
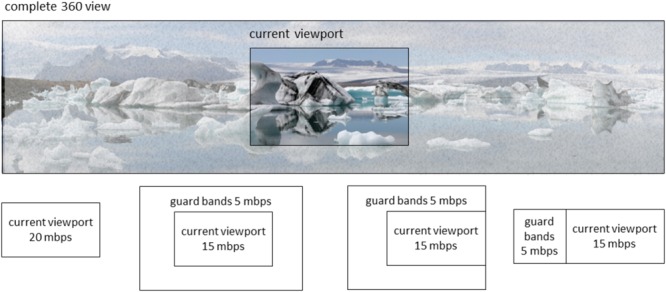
Schematic representation of possible streaming decisions in terms of spatial area and mbps (megabit per second) to present images to a user of a VR headset. The lower insets, respectively, represent possible decisions in cases that the head is not expected to rotate; expected to rotate in an unknown direction; expected to rotate in some leftward direction; and expected to precisely rotate to the left.

**FIGURE 2:**
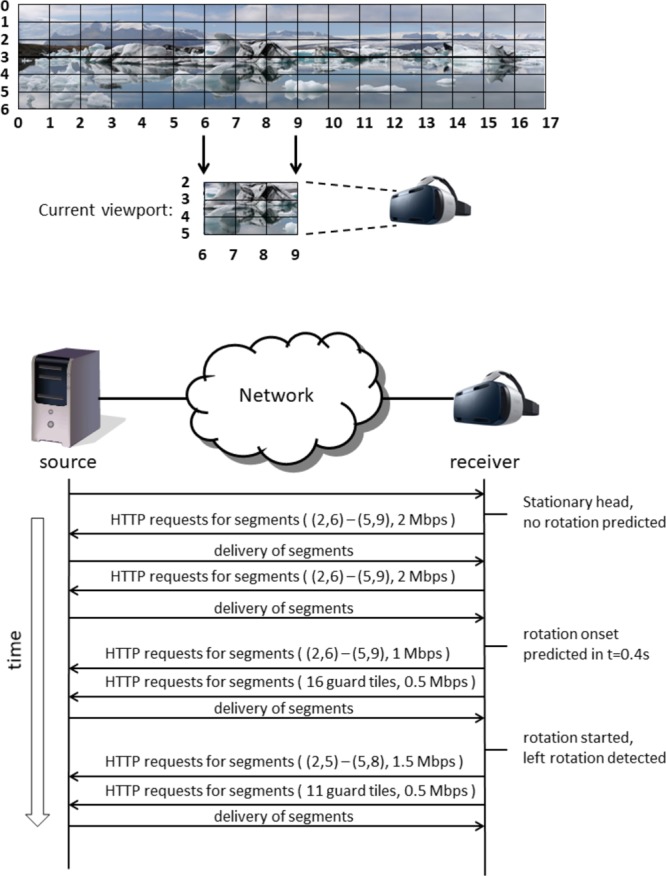
Schematic representation of a streaming process over time, integrating prediction, and detection of head rotation.

The problem with FoV-based VR streaming approaches is that new content needs to be made available quickly when the VR user rotates his or her head. Currently, such rotations are detected using one or more sensors in or near the VR headset, such as a gyroscope or an external camera facing the VR user. With video streaming, it will take some time before a new video can be started, because of the delays inherent in video streaming (e.g., requesting delays, encoding delays, network delays, buffering delays, decoding delays). When requests for a new part of the video are sent only after the head rotation is detected, new video material for the new viewing direction will only be available after some time. In real-time situations, for example in VR video conferencing, the lowest achievable end-to-end delay (from camera glass to display glass) is in the order of 100 ms (Kegel et al., 2012). Lower delay comes at the cost of spatial quality. In nonreal-time situations, for example, watching a TV broadcast, encoding is already performed, and the complete video can be made available on a node in the network close to the VR user. In such, and otherwise optimal (i.e., lab) circumstances, delays as low as 50 ms may be possible. These ultra-low delays will come at the cost of higher bandwidth usage, not available to the ordinary consumer. A potential solution is to optimize FoV-based streaming approaches in terms of latency and content quality. This can be dealt with by streaming the current field of view in high resolution (i.e., using most bandwidth for this part) and stream some of the adjacent parts in lower resolution (i.e., stream “guard bands” using some bandwidth). If we can predict when and where the head is going to rotate, appropriate adjacent parts can be streamed (only) at appropriate times, such as to optimize what to spend the bandwidth on. The specific way that this can be done depends on what can be predicted and how precise. **Figure 1** illustrates this schematically. If it can be predicted whether the head is likely to rotate or not, only the current view needs to be streamed if the head is likely to remain stationary; if the head is likely to rotate, guard bands completely surrounding the current view can be streamed in addition to the current view. If it can be predicted that the head is likely to rotate to the left, a larger or a smaller and more specific guard band to the left can be streamed, depending on the precision with which the exact rotation of the head can be predicted (examples are schematically indicated in the lower panels of **Figure 1**). **Figure 2** shows an example streaming process for a receiver and a source connected via a network, where 20 Mbps of bandwidth is available for the receiver for this streaming process, and in the case that only rotation onset can be predicted without knowing the rotation direction. In this example, the user initially sits still and looks in one direction. Normally, the streaming process should always have some way to deal with a sudden onset of movement. This may consist of a low-resolution fallback layer continuously provided for the entire image, or of the delivery of guard band tiles surrounding the current viewport. However, since here it is predicted that the head will remain stationary, this is not necessary, and only the tiles for the current viewport are requested. Thus, the best image quality can be attained given the current bandwidth. In this case, for all tiles in the current viewport segments are retrieved at the highest quality, that is, at 2 Mbps. As long as no movement onset is predicted, the receiver can continue to request new segments for these same tiles, as conceptually shown in the figure by repeating the same HTTP request. Then, at a certain point in time, rotation onset is predicted to occur in 400 ms, giving the receiver ample time to retrieve segments for new tiles. Because in this example, only rotation onset is predicted and not rotation direction, guard band tiles are requested next to all current viewport tiles. The streaming is limited in bandwidth to 20 Mbps, which in this situation means that the viewport quality will decrease to make bandwidth available for retrieval of guard band tile segments. This is shown in **Figure 2** in the next step, where the segments for the nine viewport tiles are now requested at 1 Mbps and the 16 guard band tiles surrounding the viewport are requested at 0.5 Mbps. Then, the actual rotation starts as detected using a motion sensor such as a gyroscope. This sensor also detects the rotation direction. Once the direction is known, the streaming can be further optimized by requesting only guard band tiles in certain directions, that is, in this case to the left, up, and down, as a complete reversal of head movement from left to right cannot happen instantaneously. Finally, in the last two HTTP requests, movement has already started, the viewport has moved one tile to the left and only 11 guard band tiles (i.e., left, up, and down) are still requested. For more details on this example, as well as other examples, see Schilt et al. (2016).

We propose to use EEG brain signals to predict rotation onset and rotation direction in order to optimize the FoV-based streaming approaches (Brouwer et al., 2017). Before a body movement takes place, several processes have occurred in the brain. Depending on what elicited the movement, or what is its goal, attention has been drawn, a decision has been taken, and the movement has been planned. After planning, signals are sent to the muscles to contract, and only then the movement starts. This means that we can potentially use brain signals to shorten the time of reliably detecting movement onset compared to conventional methods, or to even predict the movement.

The literature reports two general signals related to movement planning that can be captured by EEG. One is the readiness potential (cf. lateralized readiness potential, contingent negative variation, or CNV, bereitschaftspotential: Walter et al., 1964; Kornhuber and Deecke, 1965; Coles, 1989; Leuthold et al., 2004; Guggisberg and Mottaz, 2013), and the other is (lateralized) event related desynchronization (Pfurtscheller, 2001). The first type of signal has been observed at the motor cortex when signals are synchronized on (hand) movement onsets. Depending on the exact research paradigm, a slow negativity can start to occur already 2 s before movement onset. This effect has been attributed to nonspecific (attention related) preparation for action. Around 400 ms before movement onset, the signals become asymmetric according to whether the movement is left or right (the “lateralized” part). For the desynchronization type of signal, we do not examine EEG waves as a time-series signal as we do for the readiness potential, but we look at the power in the 10–12 Hz (alpha or mu) frequency band. A desynchronized signal, represented by a low power in the 10–12 Hz band, roughly corresponds to a high level of activation of that area. Left hand movement imaging, planning, and execution correspond to a relatively low power in the right hemisphere, and vice versa. Studies on these signals usually employ hand or arm movements. In the literature, we could not find specific information about EEG and head rotation. Still, similar information from EEG as mentioned above may be used.

In order to be able to use brain signals to predict a single movement, it does not suffice to look at signals averaged across many instances of, for instance, left-, right-, and no movements, even though this is the common approach in research such as cited above in order to average out noise. For our type of application, we will have to be able to extract this information reliably from a single, short interval of brain data. There has been successful work in this area with respect to (offline) predicting single movements in the case of emergency braking in virtual or real driving (i.e., predicting movement of the foot or leg before it is detectable from letting go of the gas pedal: Haufe et al., 2011, 2014; Kim et al., 2015); steering a steering wheel in virtual driving (Gheorghe et al., 2013), self-paced reaching movements (Lew et al., 2012), and self-paced foot movements (Liu et al., 2017). These studies show that EEG allows predicting movement onset 200 to 800 ms before it is detected using conventional measures and/or electrical signals from the muscles (EMG – Lew et al., 2012; Haufe et al., 2014).

As of yet it is unknown whether such single trial movement prediction is possible for the case of head rotation. When head rotation is elicited by the occurrence of relevant visual or auditory stimuli (such as emergence braking was elicited by the perception of braking lights in the study referred to above), brain signals reflecting the perception of and attention to these events may be exploited to predict body movement. In such cases, automatic detection of or otherwise knowing about these events may also be used directly to predict head rotation, decreasing the chances of EEG to be of added value in the prediction. Therefore, we are especially interested in the case of predicting voluntary, completely top-down determined head rotation. This represents a different, relatively hard case since rotating the head involves a large number of muscles on both sides of the body, a relatively small amount of motor cortex is dedicated to the neck, and, as mentioned, we cannot make use of brain processes associated with processing sensory signals. On the other hand, other markers such as those related to spatial attention are expected to be especially tightly connected to head movements compared to limb movements, and may be used. Additionally, in this case of voluntary, top-down movements, other higher order processes of attention and planning may be used as well.

In the current study, we investigate whether it is possible to predict (the direction of) a single, voluntary head rotation, and if so, how long in advance and how accurately this can be done for a (simulated) real-time scenario. This is important given our envisioned VR use case, where a continuous stream of EEG would need to be judged continuously with respect to the likelihood of upcoming head rotations. For rotation direction, we focus on left- and rightward direction since the horizontal dimension is usually the one with the largest changes in displayed imagery. We further focus here on features from EEG time-series rather than frequency analysis since single trial movement prediction studies showed that time-series based features are strongly preferred over frequency based features (Lew et al., 2012; Haufe et al., 2014; and especially Liu et al., 2017).

## Materials and Methods

### Participants

We recorded from 11 participants who were recruited through the local participant pool of the research institute where the study was conducted. They were between 20 and 60 years old (SD 12.6). This study was carried out in accordance with the recommendations of the Helsinki Declaration of 1975, as revised in 2013 (World Medical Association, 2013). The protocol was approved by the TNO Institutional Review Board (TCPE). All subjects gave written informed consent in accordance with the Declaration of Helsinki. Participants received monetary compensation for their time and travel.

### Equipment

For EEG, 32 active silver–chloride EEG electrodes were placed according to the 10–20 system and were referenced to the Common Mode Sense (CMS) active electrode and Driven Right Leg (DRL) passive electrode (Biosemi ActiveTwoMk II system). Participants wore a light-weight Head Mounted Display (HMD) (FAT SHARK Dominator HD2). This HMD contains an inertial measurement unit (IMU), combining signals from gyroscope, accelerometer, and magneto sensors. The 128 Hz IMU output was used to collect data on the head’s actual rotation.

### Procedure

After the study was explained, any questions answered and the informed consent forms signed, the EEG sensors were attached and the HMD put in place. Participants were asked to make self-paced right- and leftward head rotations, starting from and returning to the center at voluntarily chosen, arbitrary times, but leaving at least 2 s in between rotations starting from the center. The HMD showed a black screen since as explained in the introduction, we here test the situation that head rotations are performed voluntarily, that is, we did not want to capitalize on brain signals that are expected to be generated by perceiving and attending to visual or auditory stimuli. Participants were asked to perform the task for 20 min, keeping their eyes open. Then the HMD was taken off for a 10-min break after which another 20-min session followed.

### Analysis

#### Extracting Current Head Rotation

**FIGURE 3:**
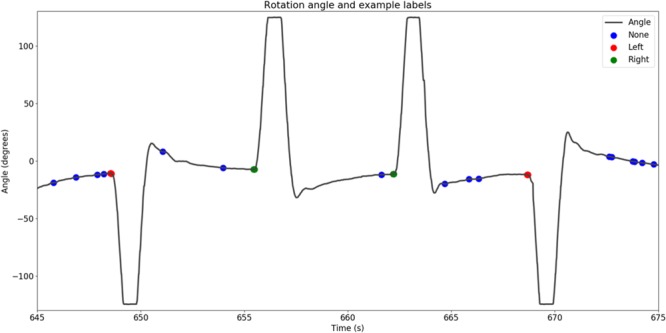
Example IMU data showing the extracted movement onsets, as well as a random sample of moments in time that were considered to be no movement.

We moved a sliding window of 125 ms over the head rotation velocity data of each participant. When the velocity exceeded a noise threshold and remained there, we defined the moment that the velocity exceeded the threshold first as movement onset. The noise threshold was determined by the variance of the velocity when the participant kept his or her head steady. This procedure resulted in the labeling of each frame of corresponding EEG data as “no rotation,” “leftward rotation,” and “rightward rotation.” **Figure 3** shows example IMU data with corresponding rotation labels.

#### Preprocessing EEG

When selecting data intervals for training the model, intervals containing EEG with amplitudes over 80 mV were discarded as noise. Since we are particularly interested in slow components, EEG was band pass filtered between 0.75 to 8 Hz. Finally, EEG was downsampled to 128 Hz. The same preprocessing was done when validating and testing the model.

#### Training and Testing the Neural Network

For each participant, a multi-layer perceptron model (Sarle, 1997; Heaton, 2005) predicting head rotation was trained and validated. Similar to what was done in previous studies (e.g., Huan and Palaniappan, 2004; Mirghasemi et al., 2006; Nakayama et al., 2007; Manyakov et al., 2011), we used a dense neural network with three hidden layers (512, 256, and 6 nodes, respectively). All hidden layers used the ReLu activation function (Hahnloser et al., 2000) and L2 regularization. The first two hidden layers also used 10% dropout (Srivastava et al., 2014). The input layer received all 1024 features, and the output layer contained three nodes with a sigmoid activation function for each of the three classes. The network was trained for 150 epochs with the ADAM optimizer (Kingma and Ba, 2014) in batches of 150 samples, which were normalized. The learning rate was 0.001 with a categorical cross-entropy loss function.

**FIGURE 4:**
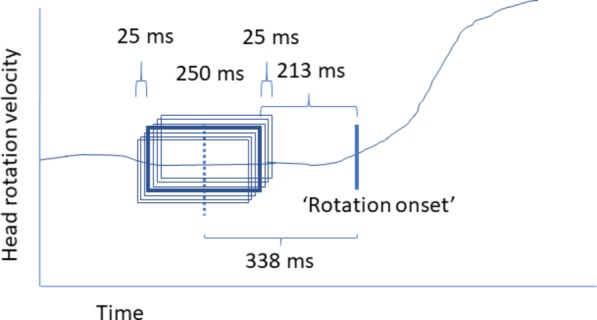
Schematic overview of defining head rotation intervals for training the model.

For training the model, 250 ms intervals of 32 electrode EEG data were labeled as preceding no rotation, preceding leftward rotation, and preceding rightward rotation. Only head rotations going from the center (facing straight ahead) to one of the sides were considered since in contrast to movements started with the head facing to one of the two directions, the rotation direction of the next movement is unknown. Each rotation was associated with seven partially overlapping, jittered windows, where the window closest to the rotation onset ended 188 ms before rotation onset as defined by the algorithm extracting head rotation as outlined above, and the window furthest away from rotation onset started 488 ms before rotation onset. The center of the center interval was 338 ms before rotation onset. **Figure 4** provides a schematic illustration of this. The width of these windows and their positioning in time may not be optimal; however, they fit to the effort to stay clear of actual head rotation while allowing picking up a differential signal. We estimated that differences between no rotation, left-, and rightward rotation would start to occur around 300–400 ms given the previous single trial movement prediction studies, as well as that in our task (rotate the head self-paced, leaving at least 2 s in between) participants were not expected to plan a movement very long ahead of time. The width of the window needed to be long enough to be able to deal with potential timing differences in onset, and short enough to be able to have a clear separation between “no rotation” intervals and rotation intervals, while also staying clear of head rotation. “No rotation” intervals never overlapped with periods of head rotation (as defined above) and always ended at least 1000 ms before rotation onset.

For each participant, an equal number of intervals for right, left, and no rotation was included in training and testing the model where this number was determined by the smallest number of right- or left-rotation intervals. Randomly chosen intervals were left out from the other categories in order to obtain equal numbers. On average, participants’ datasets consisted of 975 intervals in each class for training (SD 137; participant range between 816 and 1244) and 110 intervals in each class for testing (SD 23; participant range between 81 and 152). Because of the jittered windows, these numbers represent seven times the number of used rotations.

The model was trained and tested in epochs. Every epoch, 72% of data from the first and 72% of data from the second 20-min block was selected as training data; 18% was selected as validation data to optimize the network; and 10% (a 4-min continuous stream of data) was set aside as test data to determine the accuracy of the final model.

Every epoch, weights of the neural network were adjusted such as to fit the training data to the labels. The output of the model was a three-number vector indicating the probability of a label as belonging to none, left or right, for example, [0.003; 0.025; 0.002]. This was compared to the vector representing the truth, for example, for a leftward rotation [0; 1; 0]. In this example, the model would be accurate (the highest probability corresponds to the correct label) but since the probabilities are all quite low (0.025 is far from 1), the loss (root mean squared error) would be high. The model optimized on limiting the loss (categorical cross entropy). Then, the final trained model was applied to the withheld test set in order to monitor generalization of classification performance to completely unseen data.

Given the stochastic nature of the modeling, this procedure was repeated three times for each participant, such that three values were obtained of the final model’s performance on the unseen data.

#### Applying the Trained Model

As described above, the model was trained and tested on labeled intervals of data. However, in our real-time application scenario, we would need to classify streaming data rather than predefined intervals of data. This was simulated by having the model classify the withheld 4-min stream of test data by presenting it as subsequent 250 ms intervals of data, every time shifted by one frame (i.e., 7.8 ms).

## Results

**FIGURE 5:**
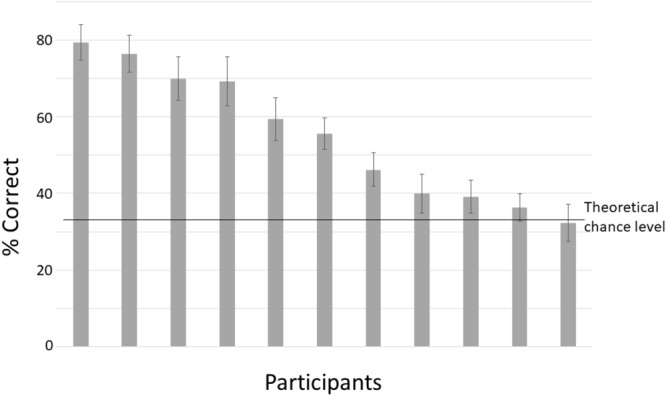
Mean classification accuracy for the testing set, separately for each participant. Chance level is 33%. Error bars denote standard deviations.

**Figure 5** shows classification accuracy for each participant for the withheld test data. The percentage correct indicates the percentage of intervals that is correctly classified into one of the three different classes (right, left, and no rotation). For most participants, classification accuracy is above chance level. The upper level of chance performance is around 38% (110 trials – Müller-Putz et al., 2008).

**FIGURE 6:**
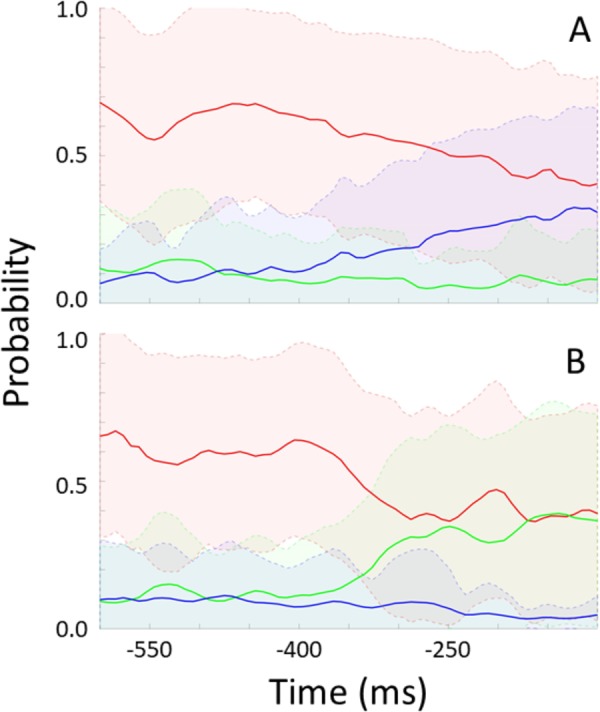
Modeled probability for no rotation (red), rightward rotation (blue), and leftward rotation (green) over time preceding rightward rotation **(A)** and leftward rotation **(B)**. Probabilities are averaged across participants and data intervals [representing a total of 58 rightward rotations in **(A)**, and a total of 63 leftward rotations in **(B)**]. Shaded areas represent standard deviations.

**FIGURE 7:**
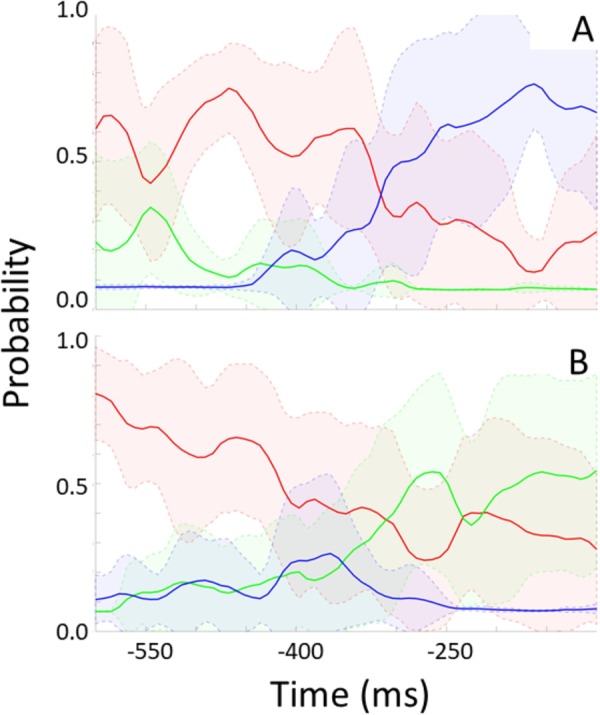
Modeled probability for no rotation (red), rightward rotation (blue), and leftward rotation (green) over time preceding rightward rotation **(A)** and leftward rotation **(B)** for one participant. This participant produced eight rightward head rotations in the test data [i.e., **(A)** represents eight rightward rotations] and 12 leftward rotations [i.e., **(B)** represents 12 leftward rotations]. Shaded areas represent standard deviations.

For each participant, the trained model was applied to unseen “streamed” test data to simulate a real time situation. For every frame, the model indicates the probability of no rotation, left-, and rightward rotation. **Figure 6** shows these probabilities over time preceding rotation onset (time 0) and averaged across epochs and participants. **Figure 6A** shows data preceding rightward head rotation and **Figure 6B** leftward rotation. **Figure 7** represents the same but for only one individual participant (the one with the highest classification accuracy for the test set as represented by the left-most bar in **Figure 5**). Standard deviations are indicated by the shaded areas. The graphs show that 600 ms before rotation onset, the probability of no rotation is high whereas the probabilities of right- and leftward rotations are both low, and equally low. Around 400 ms before rotation onset, the probability of no rotation starts to decrease. Shortly before, the probability of the rotation in the correct direction starts to increase. Notably, and consistent with the observation that low accuracy in **Figure 5** is mostly due to intervals being incorrectly classified as “no rotation” intervals, the probability of no rotation remains high. The figures showing data from the participant that can be classified relatively well differentiate from the overall average in that the “no rotation” probability decreases steeper and the probability for the correct rotation increases steeper such that 330–200 ms before rotation onset, the probability of the correct rotation is on average higher than the probability of no rotation probability.

**FIGURE 8:**
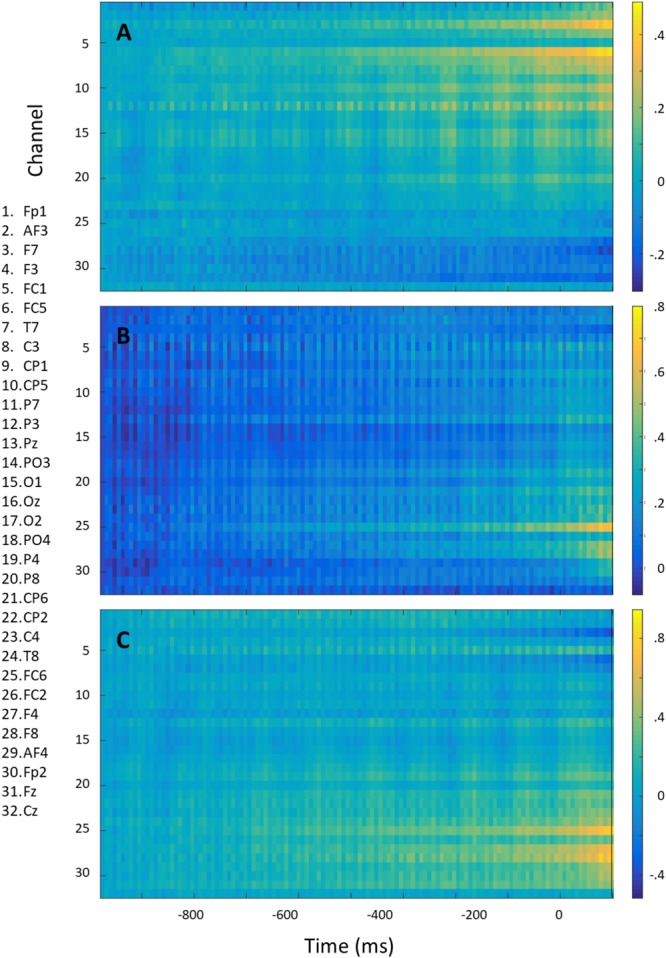
Mean voltage over the 1000 ms preceding right **(A)** and left **(B)** rotation onset and their difference (left minus right) **(C)** for each electrode. Epochs were baselined on –1000 ms.

**Figure 8** shows the mean voltage over the 1000 ms preceding right (A) and left (B) rotation onset for each electrode (baselined on the start of the epoch). The difference signal is shown in **Figure 8C**. For this figure, equal numbers of right- and left rotations were used for each participant, and averaging occurred first per participant and then across participants so that each participant is represented equally strongly in the figure. The information distinguishing between the two directions of rotation is mostly frontal and lateralized. There seems to be no information at the electrodes close to the neck.

## Discussion

We found that whether, and in which direction a head is going to rotate can be predicted on the basis of EEG data starting at around 400 ms before the rotation occurs. Performance is variable between participants, where the main difference seems to be in the strength of the bias to label data as preceding “no rotation.” Intervals of unseen test data could be classified as belonging to one of the three rotation categories with accuracies ranging between 32% (chance level) to 79%.

Whereas we could have expected the best prediction around the timepoint at which the intervals were selected for training the model (i.e., at 338 ms before rotation onset), after which a decrease may have set in, we do not observe such a “tuning.” At around 450 ms before rotation onset, the probability of the correct direction of head rotation starts to rise, where it keeps rising or stays at the same level until the rotation is made. The model thus bases itself on processes that last until rotation onset rather than processes that occur in a bounded interval before rotation onset and is not hindered by processes that start occurring closer to the rotation onset.

We did not think that eye movements could be very helpful in predicting head rotation, and that our models could capitalize on artifacts generated by eye movements preceding head rotation, since normally, top-down large head rotations are not preceded by eye movements. Freedman (2008) reviews studies on eye-head coordination. When eyes and head are free to move, they start moving at around the same time, with the eyes arriving at the desired gaze location before the head stops moving. Freedman reports a few studies where eye movements precede head movements, but this was with (only) 30 ms. Summarizing all reviewed studies, he states that motor commands of head movement generally precede those of eye movements, and that especially for large amplitude movements (as in our case) head movements begin well before saccades. In accordance with this, Solman et al. (2017) shows and reviews evidence that especially when gaze shifts are intentional (as in our case) rather than reacting to an appearing visual stimulus, the head rather leads the eyes than the other way around. However, given the frontal lateralized results shown in **Figure 8**, it is important to further examine the role of eye movements. While EOG electrodes are difficult to combine with wearing a HMD, future experiments should use a HMD with an integrated eye tracker to test this. For the application, the underlying cause of the signal is relevant insofar that it may predict whether or not the model will generalize to other situations, and to explore other data streams (in this case, an embedded eye tracker) that may add to or replace the information obtained from EEG electrodes.

Given the “no rotation” probability bias (**Figure 6**), it would be suboptimal to base a VR data-streaming decision on the highest probability of one of the three classes at some point in time, as was done to determine the classification accuracy represented in **Figure 5**. An algorithm aiding such a decision should base the decision on whether and which type of rotation is expected on the consistency with which one of probabilities of the two directions of rotation diverges from the other, together with a decrease in the no rotation probability. Note that for the participant presented in **Figure 7**, the model is very certain of the low probability of the wrong direction of rotation (very small standard deviation) from 250 ms before movement onset. This would support a good VR data streaming decision.

We should note that we presented the probability data as a function of time such that it makes sense from the point of view of predictive value contained in the EEG data at around that time. That is, the probabilities of the three classes resulting from an interval of 250 ms of EEG data are plotted at the time of the center of that interval. The information is only available for use after the whole interval has elapsed, that is, in this case where we used 250 ms intervals, the information is available 125 ms later than plotted in **Figures 3, 4**. Still, our data show that the EEG-based predictions can be available well in advance of the actual head rotation, and early enough for enhancing VR experience. Applying a trained model to classify incoming streamed data takes a negligible amount of time and bandwidth (Balakrishnan and Puthusserypady, 2005; Abadi et al., 2016).

While in this study, we used gelled EEG electrodes, meant for laboratory use, wearable “dry” or water-based EEG electrodes are already available and shown to be able to genuinely detect brain activity (Barham et al., 2017; Krigolson et al., 2017). Such electrodes could be relatively easily integrated in a VR headset so that users would not need to wear an extra device. Other types of information coming from such integrated EEG electrodes, notably information reflecting affective or cognitive state, may be exploited as well, for example, in the context of gaming (Nijholt et al., 2009). Integrated electrodes in a device that individuals wear anyway potentially allows for collecting large amounts of data that may enable modeling independent of the individual that is otherwise difficult to do (Solon et al., 2017).

There are several routes to improve the classification accuracy of the models. Classification accuracies around 95% for the validation data for all participants indicated that overfitting occurred, which may be dealt with using a smaller set of features or different models. Furthermore, a soft-labeling approach may be helpful. Currently, intervals of data are labeled as either preceding a (certain) movement or not whereas in some of the (earlier) windows preceding rotation there may actually not be information present in the EEG data yet. Related to this is the optimal time interval for predicting rotation onsets. In the current study, the intervals were chosen such as to maintain a solid buffer between detected rotation onset and the used interval, to prevent using signals due to actual movement that may not have reached the velocity detection threshold for determining rotation onset. However, this may not have been the optimal choice.

A general helpful property of the proposed BCI presented here that we have not exploited, is that the model can keep track of its own performance on the fly without requiring user input. It predicts a future situation – and receives information on whether this prediction was correct or not. This potentially enables adaptive optimization of the model and can aid decisions on whether or not the model is good enough to base certain decisions on. During usage, the set of data to train models on will grow automatically. While in this study, we started with head rotations that are completely voluntarily determined, without visual stimuli drawing attention, in real use the HMD will display images. Depending on the context, for example, whether the user is watching a live tennis game or is engaged in a group meeting, head rotations will be more or less strongly determined by the visual stimuli. This will affect EEG, and likely also the specific signals associated with left-, right-, and no rotation which may well be exploited. It may prove helpful to build and/or improve models separately for different contexts. In addition, and depending on the context, other features that are predictive of head rotations, directly acquired from the presented visual and auditory stimuli, can be exploited in the model and improve predictions.

## Conclusion

In sum, we showed the feasibility of predicting single voluntary, top-down determined head rotations in a simulated online scenario and indicated how predicted head rotations could be used to improve streaming images to a headset. The proposed BCI has the potential of large scale application given that users would not need to wear additional equipment on the head, and given that the BCI can train and validate itself on the fly. If the predictions are not deemed good enough, the VR system will function in its default way; if the model becomes better, presentation of imagery can be improved in ways as indicated by the examples in **Figures 1, 2**. In addition to the ability to monitor its own errors, consequences of errors are not grave – errors can cause a nonoptimal VR experience but will not result in dangerous situations as could be the case when BCIs are used for instance in vehicle control. All of this makes the costs of using the BCI in real life relatively low. The potential gain is an improved VR experience. Work that needs to be done before the proposed BCI is a fact includes, as indicated above, testing head rotation prediction in different other types of scenarios including visual and auditory stimuli (where the BCI may exploit neural signatures associated with detection of and attention to these stimuli; and where these stimuli may be used in the prediction directly), and determining the exact algorithm of what exact streaming decision to make what the incoming information. Importantly, good VR data streaming decisions not only depend on what exactly can be predicted with what certainty, but also on the net-effect of these decisions on user experience. The best trade-off (for a certain user, in a certain context) with respect to the number or length of noticeable delays and the degree of (changes in) spatial resolution needs to be determined. If headsets with embedded BCIs to predict head rotation are used regularly, this will produce a large and potentially valuable data base, not only for improving the BCI itself, but also potentially valuable from a general neuroscientific point of view. EEG data together with the presented stimuli and behavioral data may prove valuable in studying attention and motor planning processes in ecological circumstances, or even studying real-life cognitive and affective state.

## Author Contributions

AB and HS contributed to the conception and design of the study. JV performed the analysis and strongly contributed to its design. AB wrote the first draft of the manuscript. JV and HS wrote sections of the manuscript on, respectively, the analysis and data streaming. All authors contributed to manuscript revision, read, and approved the submitted version.

## Conflict of Interest Statement

The authors declare that the research was conducted in the absence of any commercial or financial relationships that could be construed as a potential conflict of interest.
